# Targeting Sensory Neuropathies Through Neurotrophin-Based Approaches

**DOI:** 10.1007/s10571-026-01756-0

**Published:** 2026-06-08

**Authors:** Veronica Mohamed Hizam, Di Prisco Stefania, Anna Pisani, Boccella Serena, Brandolini Laura, Claudio Grassi, Fabiola Paciello, Anna Rita Fetoni

**Affiliations:** 1 Department of Head and Neck Surgery, Università Cattolica del Sacro Cuore, Rome, 00168 Italy; 2 Research & Early Development (R&D), Dompé Farmaceutici SpA, 80145 Naples, Italy; 3 Department of Neuroscience, Unit of Audiology, Università degli Studi di Napoli Federico II, Via Sergio Pansini, 5, Naples, 80131 Italy; 4 R&D, Dompé Farmaceutici SpA, L’Aquila, Italy; 5 Department of Neuroscience, Università Cattolica del Sacro Cuore, Rome, 00168 Italy; 6 Fondazione Policlinico Universitario A. Gemelli IRCCS, Rome, 00168 Italy

**Keywords:** Neurotrophins, Sensory neuropathies, Neuroprotection, Therapy

## Abstract

Neurotrophins, including NGF, BDNF, and NT-3, are crucial regulators of neuronal survival, synaptic plasticity, and regeneration in sensory systems. Emerging evidence suggests that neurotrophin signaling may represent a common biological axis underlying both sensory neuron vulnerability and repair across auditory, visual, and olfactory systems. This review examines the roles of neurotrophins in maintaining neuronal integrity, promoting synaptic repair, and facilitating functional recovery in sensory pathways. We delved into the molecular mechanisms by which neurotrophins influence neuronal survival and plasticity, with a particular focus on cochlear synaptopathy, optic neuropathy, and olfactory dysfunction. Additionally, we compared neurotrophin-based therapies to emerging approaches, such as gene therapy, small molecules, and cell-based treatments, emphasizing their potential to complement and enhance existing strategies. Despite their therapeutic promise, several challenges persist in translating neurotrophin therapies to clinical practice, including issues related to delivery efficiency and pharmacokinetics. Overall, neurotrophin-based interventions, especially when integrated with complementary regenerative approaches, may offer an effective strategy for restoring sensory function and modifying disease progression, paving the way for future clinical applications.

**Graphical Abstract:**

**Figure:**
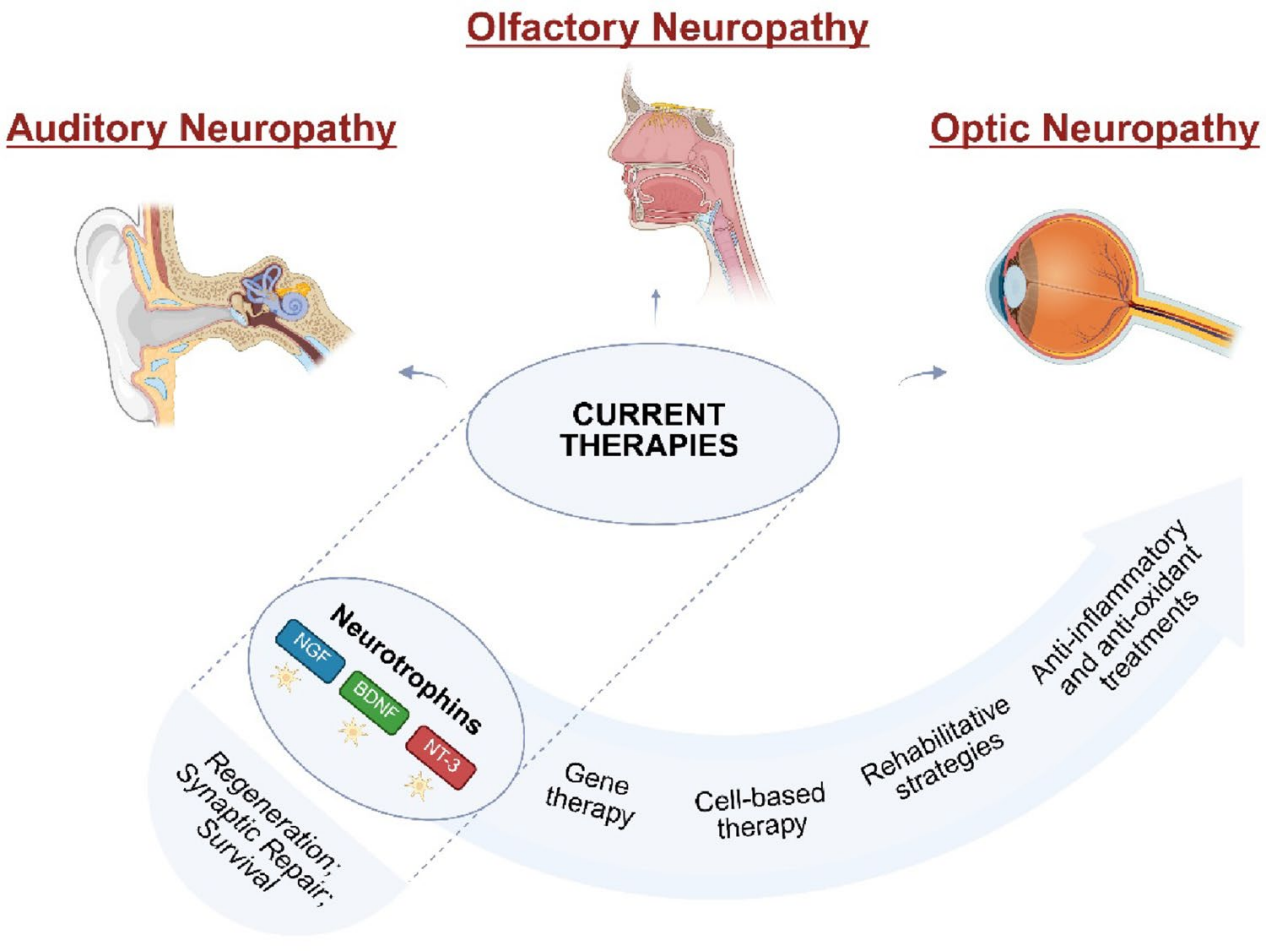

Overview of current and emerging therapeutic strategies for sensory neuropathies. Representation of current and emerging therapies for sensory neuropathies, including auditory, olfactory and optic neuropathies. Neurotrophins are highlighted as central modulators of neuronal survival, synaptic repair, and regeneration, alongside gene and cell-based therapies, rehabilitation, and anti-inflammatory/antioxidant approaches, within a multimodal strategy to preserve neuronal integrity and restore sensory function.

## Introduction

The peripheral nervous system (PNS) constitutes a crucial communication network linking the central nervous system (CNS) to the rest of the body, thereby enabling both sensory perception and motor control (National Cancer Institute; Koop and Tadi, 2023). Among the twelve cranial nerves (CN) of the PNS, several play essential sensory roles, mediating modalities such as vision, hearing, taste, smell, and touch (Cleveland Clinic, 2024). The olfactory (CN I) and optic (CN II) nerves are unique among the cranial nerves, as they are considered direct extensions of the CNS. The optic nerve is myelinated by oligodendrocytes, whereas olfactory axons are unmyelinated and are supported by specialized olfactory ensheathing cells rather than Schwann cells (Rueda-Lopes, 2021).

Sensory neuropathies affecting the auditory, visual, and olfactory systems arise from a wide range of etiologies, including ischemic and inflammatory processes, autoimmune or infectious insults, toxic exposures, nutritional deficiencies, trauma, neurodegenerative diseases, and genetic mutations affecting neuronal or glial function. Ageing and chronic oxidative stress further contribute to the progressive degeneration of sensory neurons and synaptic connections (Kujawa and Liberman, 2009; Williams et al., 2013).

**Fig. 1:**
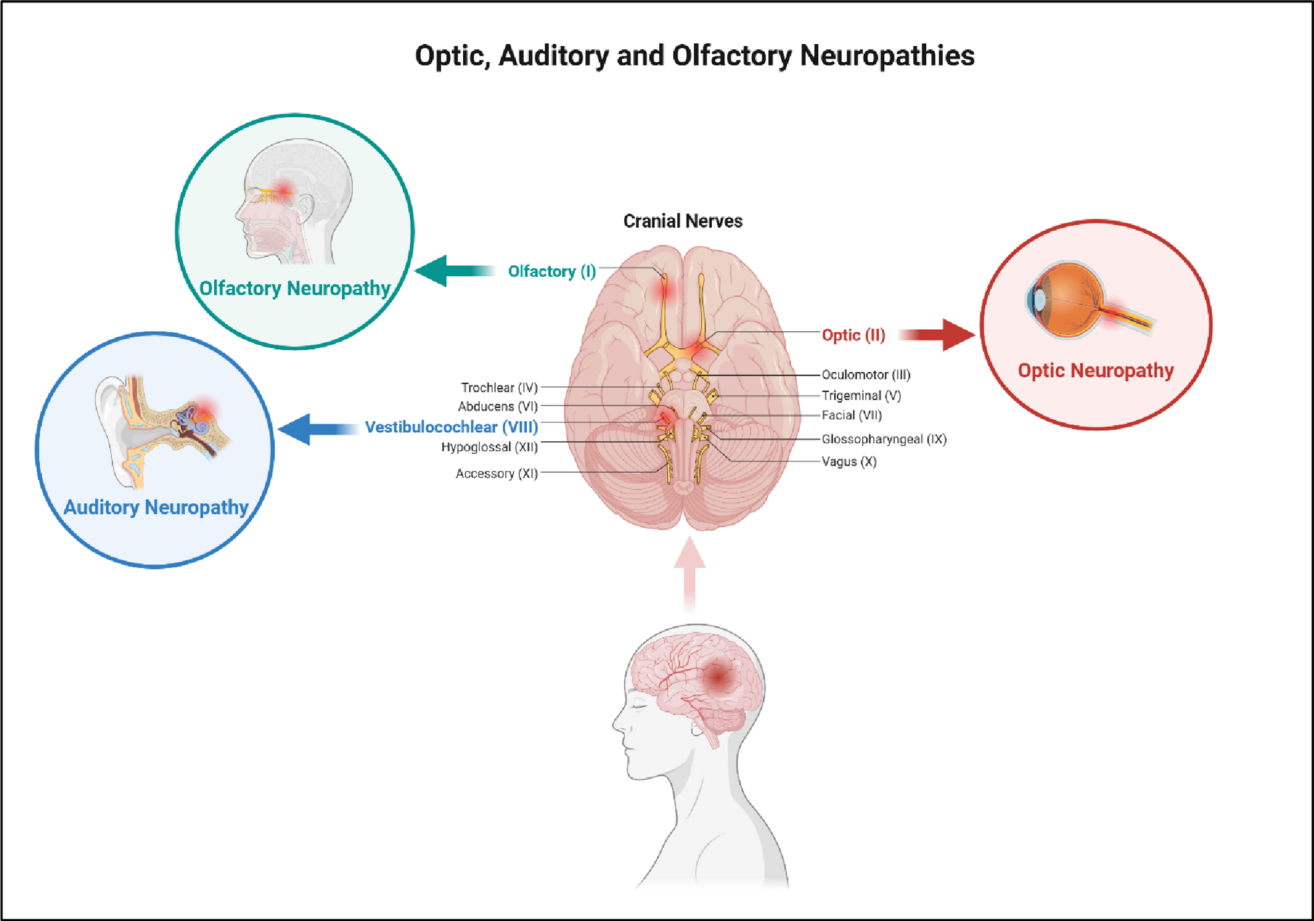
Schematic diagram of Optic, Auditory and Olfactory Neuropathies. The figure shows the cranial nerves involved in the main sensory systems: the optic nerve (II) for vision, the vestibulocochlear nerve (VIII) for hearing, and the olfactory nerve (I) for smell. The red areas indicate the sites of neuronal degeneration or dysfunction that characterize optic, auditory, and olfactory neuropathies

Damage to the optic (II), vestibulocochlear (VIII), or olfactory (I) cranial nerves results in optic, auditory, and olfactory neuropathies, respectively, as illustrated in Fig. 1.

These conditions are characterized by impaired sensory transmission and progressive neuronal degeneration. Although multifactorial in origin, they share a limited regenerative potential of the affected neural tissue, representing a significant cause of sensory disability.

Given the limited regenerative capacity of sensory neurons, developing strategies that promote neuronal survival, axonal regeneration, and synaptic repair remains a significant challenge. In this context, neurotrophins, a family of growth factors that includes Nerve Growth Factor (NGF), Brain-Derived Neurotrophic Factor (BDNF), Neurotrophin-3 (NT-3), and Neurotrophin-4 (NT-4), play a key role in the development, maintenance, and functional plasticity of the nervous system (Huang and Reichardt, 2001). These proteins regulate essential processes such as neurogenesis, synaptogenesis, and neuroprotection during both embryonic development and adulthood.

Neurotrophins exert their biological effects through high-affinity Tropomyosin Kinase receptors (TrkA, TrkB, TrkC) and the low-affinity neurotrophin receptor p75 (p75^NTR^) (Chao, 2003). Ligand binding induces dimerization and autophosphorylation of the Trk receptor, triggering intracellular signalling cascades, including the MAPK/ERK, PI3K/Akt and PLCγ pathways, which regulate neuronal survival, differentiation and plasticity (Barker et al., 2020; Wang et al., 2024). In contrast, p75^NTR^ mediates pro-apoptotic or pro-survival responses, depending on the cellular context, through signaling pathways such as the JNK-p53-Bax or NF-κB (Kaplan et al,.^2000^; Friedman and Greene, 1999).

Growing evidence indicates that dysregulation of neurotrophin signalling contributes to the pathogenesis of various sensory neuropathies, while exogenous administration or targeted modulation of these factors can promote neural repair and functional recovery. Notably, accumulating data suggest that neurotrophin signalling may represent a shared molecular axis linking neuronal vulnerability and regenerative capacity across different sensory systems. Understanding the molecular mechanisms that regulate neurotrophin activity in the auditory, olfactory, and visual systems is therefore essential not only to elucidate disease pathogenesis but also to identify common principles governing degeneration and repair in sensory pathways.

In this review article, we propose a unifying theoretical framework in which the response to neurotrophins is a key determinant of the balance between degeneration and regeneration in the various sensory systems. From this perspective, we examined the key roles of neurotrophins in the auditory, visual, and olfactory systems, focusing on the molecular mechanisms underlying their neuroprotective and regenerative actions. Neurotrophin-based approaches are compared with emerging therapeutic strategies, including gene and cell-based therapies, neuroprotective compounds, antioxidants, and anti-inflammatory agents. Finally, the review discusses current challenges, future perspectives, and potential clinical applications of neurotrophins in the treatment of sensory neuropathies.

## Neurotrophins in Auditory, Visual, and Olfactory Systems

Neurotrophins are key regulators of neuronal survival, differentiation, and synaptic plasticity throughout the nervous system (Omar et al., 2022; Huang et al., 2001). Within the sensory systems, they play essential roles in the development, maintenance, and functional integrity of the visual, auditory, and olfactory pathways. The fine balance between neurotrophin signalling and neuronal activity is critical for establishing proper sensory connectivity during development and for preserving neuronal function under both physiological and pathological conditions. Notably, although these systems differ in their regenerative capacity, they share common neurotrophin-dependent mechanisms that regulate the balance between neuronal vulnerability and repair.

Neurotrophins are fundamental to the development and maintenance of the auditory system. During cochlear development, BDNF and NT-3 are the primary neurotrophins expressed in the inner ear, ensuring the survival of spiral ganglion neurons (SGNs) and the establishment of precise cochlear innervation patterns (Johnson Chacko et al., 2017; Steinacher et al., 2024).

BDNF mRNA is transiently expressed in both inner (IHCs) and outer hair cells (OHCs) during early postnatal development but decreases in the mature cochlea, while NT-3 remains expressed in IHCs and supporting cells into adulthood (Wheeler et al., 1994; Ylikoski et al., 1993). NGF also contributes to early differentiation of auditory neurons, although its expression in the mature cochlea is limited and its role appears restricted to neuritogenesis rather than neuronal survival (Represa & Bern, 1989; Pirvola et al., 1994).

The corresponding receptors exhibit a dynamic spatiotemporal pattern: TrkA is found in HCs and in SGNs, particularly during early development (Dai et al., 2004), while TrkB and TrkC expression shifts from SGNs to HCs during maturation (Green et al., 2012). The low-affinity receptor p75^NTR^ is consistently expressed throughout inner ear development and may act as a co-receptor modulating Trk affinity and specificity (Steinacher et al., 2024). The orchestrated activity of BDNF, NT-3, and their receptors is essential for cochlear neurogenesis, synaptic refinement, and auditory neuron maintenance, highlighting their potential as therapeutic targets in auditory neuropathies and synaptopathies. However, the limited regenerative capacity of the cochlea suggests that, despite the presence of neurotrophin signaling, downstream responsiveness may be insufficient to support effective repair.

The visual system is also highly dependent on neurotrophic support for the survival and connectivity of retinal neurons and optic nerve fibres. Ocular tissues express several neurotrophins, particularly NGF, BDNF, NT-3, and NT-4, along with their respective receptors (Caminos et al., 1999). In the adult retina, Trk receptors are primarily expressed by retinal ganglion cells (RGCs), whereas p75^NTR^ is predominantly found in Müller glial cells (Hu et al., 1998). Healthy RGCs receive trophic support from Müller glial cells as well as through retrograde axonal transport from their central targets.

NGF, acting through TrkA, promotes neurite outgrowth, differentiation, and survival of RGCs during visual system development, while also mediating controlled apoptosis in specific stages of maturation. Produced locally by RGCs, bipolar neurons, and glia, NGF contributes to retinal homeostasis and plays a pivotal role in maintaining optic nerve integrity. Disruption of NGF/TrkA signalling is linked to RGC degeneration and optic neuropathies, such as glaucoma (Micera et al., 2004; Guo et al., 2020).

BDNF, via TrkB activation, supports dendritic growth, synaptic plasticity, and the maintenance of RGC function. It is synthesized in both retinal and central visual structures and undergoes bidirectional axonal transport along the optic nerve (Perez and Caminos, 1995; Weber et al., 2010). NT-3, acting through TrkC receptors on photoreceptors and Müller cells, is crucial during early retinal development, promoting neuronal differentiation and oligodendrocyte precursor proliferation in the optic nerve (von Bartheld, 1998; Shen et al., 2013). Similarly, NT-4 binds to TrkB and supports RGC survival under both developmental and degenerative conditions (Ghazi-Nouri et al., 2008; Harada et al., 2005). These neurotrophins form a complex trophic network that maintains retinal integrity and contributes to repair mechanisms following optic nerve injury.

Similar to the auditory system, the visual pathway retains a strong dependence on neurotrophin support but exhibits restricted regenerative potential, further supporting the idea that differential neurotrophin responsiveness may influence recovery outcomes.

Finally, the olfactory system is one of the few adult neural circuits with continuous neuronal turnover, making it an ideal model for studying neurotrophin-mediated neurogenesis and regeneration. Olfactory receptor neurons (ORNs), located in the olfactory epithelium (OE), undergo lifelong cycles of degeneration and renewal, regulated by neurotrophic signals derived from the OE, olfactory bulb (OBu), and olfactory ensheathing glia (OEG) (Carter and Roskams, 2002).

NGF, BDNF, NT-3, and NT-4 are all expressed in the olfactory system, albeit with distinct spatial and temporal patterns (Feron et al., 2008). NGF is mainly localized in supporting cells and promotes the regeneration and survival of developing ORNs, particularly after injury or olfactory bulbectomy (Roskams et al., 1996; Uramoto et al., 1998). BDNF is expressed in the olfactory bulb, especially in periglomerular and granule cell layers, and is downregulated with aging and in neurodegenerative diseases, suggesting a role in olfactory dysfunction (Pisani et al., 2023). Experimental studies indicate that BDNF expression in the OE increases during the early stages of regeneration and subsequently rises in the OBu, suggesting stage-specific roles in ORN renewal (Uranagase et al., 2012).

NT-3, through TrkC receptors on mature ORNs, regulates neuronal maturation and homeostasis, while TrkA and TrkB are expressed in basal and sensory neuronal layers, respectively, mediating regeneration after injury (Miwa et al., 1998; Roskams et al., 1996). The role of p75^NTR^ in the olfactory bulb remains less clear, though it may participate in developmental pruning and activity-dependent plasticity (Gómez-Pinilla et al., 1989).

In contrast to the auditory and visual systems, the olfactory pathway maintains a robust regenerative capacity, providing a unique model in which sustained neurotrophin signaling effectively supports continuous neuronal renewal.

Collectively, these findings indicate that neurotrophin signaling is a conserved regulator of neuronal maintenance and repair across sensory systems, while differences in cellular responsiveness, tissue microenvironment, and intrinsic regenerative capacity determine the extent of functional recovery. In this context, modulation of neurotrophic pathways may represent a valuable approach for olfactory neuropathies.

## Treatment of Auditory Neuropathy with Neurotrophins

Among the various sensory neuropathies, auditory neuropathy, particularly cochlear synaptopathy (CS), represents a paradigmatic model to explore the neuroprotective and regenerative potential of neurotrophins. Within the conceptual framework proposed in this review, the auditory system provides a relevant example of a sensory pathway in which neurotrophin signaling is preserved, yet regenerative capacity remains limited, highlighting a potential dissociation between trophic support and effective repair.

Hearing loss is a growing global health concern, affecting individuals across all age groups and profoundly impacting communication, cognitive performance, and quality of life (Shargorodsky et al., 2010; Wilson et al., 2017; Prasad et al., 2024; GBD, 2021). Within this spectrum, CS has gained particular attention as a neuropathic condition characterized by the selective loss of synaptic connections between IHCs and auditory nerve fibers (ANFs), while preserving the structural integrity of both IHCs and OHCs (Zheng and Guan, 2018). This form of neural injury disrupts the transfer of acoustic information to the brain, triggering downstream degeneration of SGNs, the primary relay between the cochlea and central auditory pathways (Matthews and Fuchs, 2010; Rance, 2005).

Clinically, CS manifests as impaired speech perception, sound localization, and temporal resolution, even when audiometric thresholds remain within normal limits, a phenomenon known as hidden hearing loss (HHL) (Wu et al., 2021). High-threshold type I ANFs are particularly vulnerable, contributing to suprathreshold listening deficits, especially in noise (Haggerty et al., 2023). Because standard audiometry fails to capture suprathreshold deficits, advanced diagnostic approaches, such as brainstem auditory evoked potentials (BAEPs, wave I amplitude), frequency-following responses (FFR), electrocochleography (ECochG), and stapedial reflex measurements, are increasingly used to detect synaptic dysfunction (Grant et al., 2020; Bharadwaj et al., 2015; Shearer et al., 2019).

The etiological landscape of CS encompasses genetic, environmental, and age-related factors. Mutations in genes such as OTOF, CACNA1D, and SLC17A8 impair presynaptic vesicle release and neurotransmission, whereas variants in AIFM1 and DIAPH3 impair neuronal structure or postsynaptic function (Leclère and Dulon, 2023; Akil et al., 2019). Perinatal insults, including hypoxia, hyperbilirubinemia, and viral infections, can impair auditory synapses early in life. Age-related cochlear synaptopathy contributes substantially to auditory decline, even in the absence of overt noise exposure, leading to speech-in-noise deficits in older adults (Sergeyenko et al., 2013; Fernandez et al., 2015; Fetoni et al., 2011; Paciello et al., 2023). Environmental exposures, particularly acoustic trauma and ototoxic drugs, further exacerbate synaptic degeneration through oxidative stress, inflammation, and glutamate excitotoxicity (Fernandez et al., 2020; Mao and Chen, 2021; Fetoni et al., 2022a; Browne et al., 2012; Li et al., 2023; Puel et al., 1994; Henderson et al., 2006).

At the molecular level, excessive glutamate release leads to calcium overload and mitochondrial dysfunction, resulting in excessive production of reactive oxygen (ROS) and nitrogen species (RNS), activation of the NF-κB pathway, and upregulation of pro-inflammatory cytokines such as TNF-α and IL-1β (Fetoni et al., 2019a, b; Lee et al., 2020; He et al., 2016; Ohlemiller et al., 1999; Paciello et al., 2020; Paciello et al., 2021; Fridberger et al., 1998). The ensuing redox imbalance amplifies synaptic loss through apoptotic and necrotic mechanisms, while macrophage recruitment and persistent inflammatory signalling further perpetuate cochlear damage (Fetoni et al., 2022b; Frye et al., 2019; Zhang et al., 2021). These mechanisms suggest that, despite the activation of endogenous protective pathways, the cochlear microenvironment may be insufficient to sustain long-term synaptic repair, reinforcing the concept that neurotrophin responsiveness, rather than availability alone, is a critical determinant of neuronal recovery.

Given these mechanisms, therapies aimed at preserving or restoring synaptic connectivity are central to the treatment of auditory neuropathy. Cochlear implants (CIs) provide effective sound perception when HCs are damaged, but the auditory nerve remains functional. However, their efficacy diminishes in cases of severe SGN degeneration, as in chronic auditory neuropathy, where neurotrophic support becomes essential (Zheng and Liu, 2020; Oxenham and Kreft, 2014; Lin et al., 2022; Zaltz et al., 2018; Wu et al., 2023). Combining CIs with neurotrophin-based neuroprotection, particularly using BDNF, has shown synergistic effects, enhanced neuronal survival, and improved implant outcomes (Shepherd et al., 2005; Agterberg et al., 2009; Coco et al., 2007; Wefstaedt et al., 2005).

Among neurotrophins, BDNF and NT-3 are the most extensively studied in the auditory system. By binding to TrkB and TrkC receptors, respectively, they activate intracellular signaling cascades, such as PI3K/Akt and MAPK/ERK, promoting neuronal survival and synaptic plasticity while inhibiting caspase-3–dependent apoptosis (Staecker et al., 1996; Gao, 1998; Shibata et al., 2011; Suzuki et al., 2016; Maness et al., 1994; Jiao et al., 2014; Gillespie et al., 2004; Ruan et al., 1999). Although endogenous BDNF expression declines with age, TrkB receptors remain present in adult SGNs, providing a therapeutic target for exogenous BDNF delivery (Noble et al., 2011; Yu et al., 2013; West et al., 2014). Experimental studies have shown that recombinant human BDNF (rhBDNF) restores synaptic integrity, preserves afferent fibers, and improves auditory thresholds, especially in high-frequency cochlear regions where vulnerability is greatest (Gestwa et al., 1999; Wissel et al., 2006; Tan and Shepherd, 2006; Atkinson et al., 2012; Rask-Andersen et al., 2005; Adamson et al., 2002; Singer et al., 2014). These findings indicate that exogenous neurotrophin delivery can partially overcome intrinsic regenerative limitations, further emphasizing the importance of enhancing cellular responsiveness to achieve effective functional recovery.

The main challenge for BDNF-based therapy remains its short half-life and limited diffusion across the blood-labyrinth barrier (Gunewardene et al., 2022). To resolve this problem, controlled release and local delivery systems have been developed, including thermosensitive hydrogels, PLGA nanoparticles, and intratympanic or intracochlear injection methods (Kuang et al., 1999; Rathnam et al., 2019; Xu et al., 2021; Borenstein et al., 2011; Plontke et al., 2017; Min et al., 2023). Preclinical studies have demonstrated that transtympanic administration of rhBDNF-loaded hydrogels significantly reduces ototoxic and noise-induced cochlear damage by enhancing TrkB–CREB pro-survival signalling, reducing caspase-3 activation, suppressing inflammation, and promoting vascular and synaptic repair (Pisani et al., 2025; Yu et al., 2024). Gene therapy approaches using viral vectors and sustained-release formulations such as OTO-413 offer an attractive therapeutic avenue for long-term neurotrophin expression and delivery (Foster et al., 2022).

Finally, while BDNF and NT-3 have been the principal focus, emerging evidence suggests that NGF may also contribute to auditory neuroprotection. Reduced serum NGF levels have been reported in patients with sensorineural hearing loss (Salvinelli et al., 2003), and preclinical models indicate that NGF supplementation mitigates cochlear degeneration (Castelli et al., 2023; Wang et al., 2017).

Overall, these findings underscore the potential of neurotrophin-based therapies to move beyond symptom management toward true neural preservation and regeneration. Within a broader cross-sensory perspective, cochlear synaptopathy exemplifies a condition in which neurotrophin signaling alone is not sufficient to drive robust regeneration, emphasizing the need to consider system-specific constraints that modulate the balance between neuronal vulnerability and repair.

## Treatment of Optic neuropathy with Neurotrophins

Among sensory neuropathies, optic neuropathy provides a paradigmatic model in which trophic deprivation, mitochondrial dysfunction, and impaired axonal transport converge on RGC degeneration, leading to progressive optic atrophy and irreversible visual loss. The condition encompasses a broad range of etiologies, including demyelinating and inflammatory diseases, ischemic injury, trauma, toxic–nutritional or mitochondrial disorders such as Leber’s hereditary optic neuropathy (LHON), and compressive lesions (Mori et al., 2026; You et al., 2013; Behbehani, 2007). Clinically, it manifests as decreased visual acuity, altered colour vision, and visual field defects, and its diagnosis relies on clinical history and imaging modalities such as fundus examination, optical coherence tomography, and magnetic resonance imaging (Biousse et al., 2016). At the cellular level, RGC death results from excitotoxic glutamate release, trophic factor deprivation, oxidative stress, and activation of apoptotic pathways, particularly in glaucomatous damage, where elevated intraocular pressure impairs axonal transport at the optic nerve head and induces mitochondrial dysfunction (Johnson et al., 2009; Lambuk et al., 2022; Yang et al., 2020; Fudalej et al., 2021).

Neurotrophins play a pivotal role in maintaining RGC survival, preserving axonal integrity, and modulating synaptic plasticity. BDNF promotes neuronal survival and axon growth through activation of TrkB-mediated signalling, and its deficiency has been associated with the onset and progression of glaucomatous damage. Immunolocalization studies revealed TrkB accumulation at the optic nerve head during transport failure, highlighting trophic deprivation as a major cause of RGC apoptosis via JNK–BCL-2–mediated signalling and mitochondrial collapse (Johnson et al., 2009; Lambuk et al., 2022; Pietrucha-Dutczak et al., 2018). Similarly, NGF exerts dual, context-dependent effects: TrkA binding activates pro-survival cascades such as BCL-2 upregulation and caspase inhibition, while proNGF binding to p75^NTR^ promotes apoptosis, suggesting that a favorable TrkA/p75^NTR^ ratio is critical for neuroprotection (Mesentier-Louro et al., 2017; Fudalej et al., 2021; Mallone et al., 2020).

Similar to the auditory system, the visual pathway exhibits strong dependence on neurotrophic support but limited regenerative capacity, further supporting the role of differential neurotrophin responsiveness in shaping recovery outcomes.

Preclinical and clinical studies have demonstrated the therapeutic potential of neurotrophin delivery in optic neuropathy. Topical and retrobulbar administration of NGF reduces RGC loss in experimental models of glaucoma and has been shown to improve visual field and visual acuity in patients, as demonstrated with NGF eye drops at a concentration of 200 µg/mL (Lambiase et al., 2005). An 8-week Phase I/II clinical trial confirmed the safety and tolerability of recombinant human NGF (rhNGF) in progressive primary open-angle glaucoma (Beykin et al., 2022). More recently, sustained-release NGF–chitosan implants have been shown to provide long-term neurotrophic support for up to eight weeks, protecting the ventral optic nerve and promoting axonal regeneration in experimental models (Liu et al., 2025).

BDNF-based therapies have also shown neuroprotective effects, although their translation is limited by the short half-life of the molecule and poor permeability across the blood–retina barrier (Fu et al., 2019; Steuer et al., 2005). Intravitreal injection of BDNF slows RGC degeneration after optic nerve transection, though the protection remains incomplete (Mey and Thanos, 1993; Di Polo et al., 1998; Ko et al., 2001). Gene therapy approaches using adeno-associated viral vectors (AAV), such as AAV2–BDNF or AAV2–TrkB–2 A–mBDNF, enhance the efficacy and duration of neurotrophic support by counteracting TrkB downregulation and maintaining sustained signalling in the inner retina (Osborne et al., 2018; Xu et al., 2023). The combination of BDNF with its receptor thus appears more effective than either component alone, ensuring long-term protection of RGCs.

Recent advances in delivery technology have further improved the translational feasibility of neurotrophin therapy. Nanoparticle carriers, including neurotrophin-conjugated magnetic nanoparticles, allow targeted and sustained intraocular release while minimizing systemic exposure and injection frequency (Khalin et al., 2015; Giannaccini et al., 2018). These strategies address major pharmacokinetic limitations and enhance the therapeutic potential of neurotrophins for chronic optic neuropathies.

By directly promoting RGC survival, restoring retrograde trophic support, and stabilizing dendritic and axonal architecture, neurotrophin-based treatments, particularly those employing NGF and BDNF, represent the most encouraging disease-modifying strategies for optic neuropathies. When combined with controlled-release systems or gene delivery technologies, these therapies offer the potential for durable neuroprotection and functional recovery of visual pathways.

## Treatment of Olfactory Neuropathy with Neurotrophins

Olfactory neuropathy is a multifactorial disorder resulting from damage to or dysfunction of the olfactory nerve or its central pathways, leading to partial or complete loss of smell perception (Logan, 2014). Its prevalence in the general population ranges from 3% to 20% and increases with advancing age (Kondo et al., 2020). Clinically, patients may present with anosmia, parosmia, or phantosmia of variable severity (Sjölund et al., 2017).

The etiological spectrum is broad and includes head trauma, viral infections such as influenza or COVID-19, chronic inflammatory diseases including rhinosinusitis and autoimmune disorders, neurodegenerative conditions, such as Parkinson’s and Alzheimer’s diseases, exposure to neurotoxins or pollutants, metabolic dysfunction, and congenital anomalies such as Kallmann syndrome (Stuck et al., 2023; Dekeyser et al., 2024; Piura et al., 2023; Fatuzzo et al., 2023; Toth et al., 1990; Kim et al., 2021; Kulkarni et al., 2007).

Traumatic shearing of olfactory fibers at the cribriform plate and post-viral inflammatory injury represent common etiologies. Early olfactory deficits often precede the onset of motor or cognitive symptoms in neurodegenerative disorders, underscoring their potential diagnostic relevance (Velayudhan and Lovestone, 2009).

Neurotrophins are crucial regulators of olfactory neuron survival, differentiation, and regeneration. BDNF supports the turnover of olfactory receptor neurons and promotes synaptic plasticity within the olfactory bulb (Mast et al., 2012; Uranagase et al., 2012). Decreased BDNF levels, often associated with aging or neurodegeneration, have been linked to olfactory dysfunction (Nibu et al., 2001). The common BDNF Val66Met polymorphism influences olfactory performance, with carriers of the Met allele showing reduced odor detection and discrimination scores, likely due to impaired activity-dependent secretion of BDNF (Hariri et al., 2003; Hedner et al., 2010). Receptor dynamics also play a key role: following olfactory epithelial injury, expression of the low-affinity neurotrophin receptor p75^NTR^ decreases in the glomerular layer and increases in the nerve layer of the bulb, gradually normalizing during recovery, suggesting a region-specific adaptive response (Turner et al., 1998).

Experimental studies demonstrate that exogenous neurotrophins can restore olfactory structure and function. BDNF enhances neuronal survival and axonal growth not only by activating TrkB signaling but also by alleviating growth inhibition through upregulation of Lateral Olfactory Tract Usher Substance (LOTUS), an endogenous Nogo antagonist. Systemic administration of BDNF in mice increases LOTUS expression, promotes axonal regeneration, and improves olfactory performance (Matsubayashi et al., 2023). NGF application to the olfactory epithelium following axotomy accelerates neuronal regeneration, while NT-3 supports the maturation of olfactory receptor neurons, glial cells, and progenitor populations (Yasuno et al., 2000; Jezierski et al., 2001; Simpson et al., 2003).

Recent advances in drug delivery have enhanced the feasibility of sustained neurotrophin administration. The intranasal route provides direct access to the olfactory epithelium and bulb, bypassing the blood–brain barrier and achieving high local bioavailability. Biomaterial-based platforms, such as nanoparticles, thermosensitive hydrogels, and scaffold systems, prolong neurotrophin release while minimizing systemic exposure, optimizing both efficacy and safety (Yoo et al., 2022).

Beyond pharmacological delivery, cell-based and activity-dependent interventions further enhance endogenous neurotrophic signaling. Olfactory ensheathing cells (OECs) engineered to overexpress BDNF promote neurogenesis and circuit repair, whereas olfactory training (OT) stimulates expression of plasticity-related genes, including BDNF, NGF, NGFR, and GFAP, leading to measurable improvements in odor detection and discrimination (Feron et al., 2008; Kim et al., 2019). OT has been shown to outperform corticosteroid treatment in restoring olfactory function, emphasizing a neuroplastic rather than purely anti-inflammatory mechanism. Moreover, autologous transplantation of olfactory tissue, leveraging the intrinsic regenerative and trophic properties of OECs, has shown potential to promote axonal regrowth and remyelination even in other neural systems such as the optic nerve (Shkarubo et al., 2020).

In summary, these findings identify BDNF, NGF, and NT-3 as key modulators of neuronal survival, plasticity, and regeneration in olfactory neuropathy. Advances in intranasal and biomaterial-assisted delivery, along with gene- and cell-based approaches, further reinforce their translational potential. In particular, the olfactory system stands out among sensory pathways for its preserved regenerative capacity, supporting the concept that effective neurotrophin signaling within a permissive microenvironment can drive sustained neuronal renewal and functional recovery.

## Integrative Therapeutic Strategies Targeting Neurotrophin Signaling

### Auditory Neuropathy

While neurotrophins play a central role in neuronal survival and regeneration, their therapeutic efficacy is strongly influenced by the surrounding cellular and molecular environment. In this context, complementary therapeutic strategies can enhance neurotrophin signaling by modulating inflammation, oxidative stress, cellular responsiveness, and tissue repair mechanisms. Rather than acting independently, these approaches should be viewed as synergistic components of an integrated regenerative framework. These interventions aim to mitigate oxidative stress, inflammation, and excitotoxicity while actively promoting synaptic preservation and regeneration (Ma et al., 2019; Cocchiaro et al., 2022).

#### Pharmacological and Molecular Therapies: Antibodies, Small Molecules and Neuroprotective Agents

Innovative pharmacological interventions target the molecular pathways underlying cochlear synaptopathy and auditory nerve degeneration. Among these, antibody-based therapies show potential for restoring neuronal connectivity in neurodegenerative contexts, although their application in auditory neuropathy remains exploratory (Kim et al., 2023). Small-molecule therapies are being developed to promote synaptic repair and auditory cell regeneration. PIPE-505, a γ-secretase inhibitor, has been shown to enhance synaptic regeneration and improve OHC function (Kil et al., 2022). Lithium chloride has demonstrated regenerative potential by modulating GSK-3β and activating Wnt/β-catenin signalling, thereby promoting synaptic recovery (Choi et al., 2023). Additional pharmacological agents under investigation include potassium channel modulators, which enhance OHC function and preserve cochlear excitability (Chambers et al., 2017), and apoptosis inhibitors (caspase or p53 blockers) that counteract cell death following acoustic trauma (Matsui et al., 2002).

Compounds that counteract ototoxicity, such as D-methionine and sodium thiosulfate, are effective in protecting cochlear structures during therapy with aminoglycosides or cisplatin (Kros and Steyger, 2019).

**Fig. 2:**
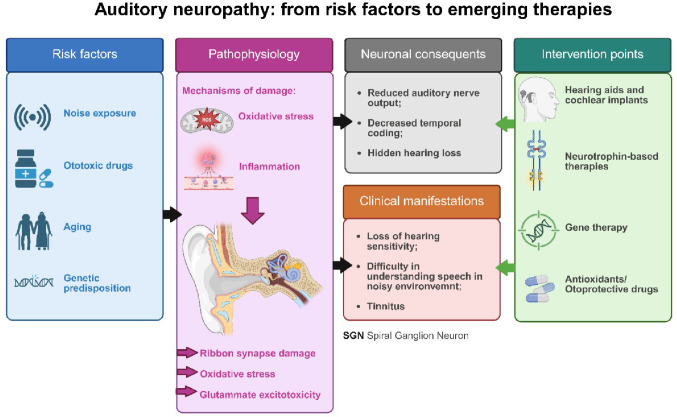
Auditory neuropathy: risk factors, pathophysiology, clinical impact, and therapeutic intervention points. Schematic overview linking major risk factors for auditory neuropathy and cochlear synaptopathy, including noise exposure, ototoxic drugs, aging, and genetic predisposition, to core pathophysiological mechanisms (oxidative stress, inflammation, and glutamate excitotoxicity). These processes drive ribbon synapse damage and SGNs degeneration, reducing auditory nerve output, degrading temporal coding, and producing hidden hearing loss despite normal thresholds. The resulting clinical manifestations include reduced hearing sensitivity, difficulty understanding speech in noise, and tinnitus. The diagram also highlights intervention points along the disease cascade: hearing aids and cochlear implants, neurotrophin-based therapies, gene therapy, and antioxidants/otoprotective drugs aimed at preserving or restoring synaptic integrity and neural function. Abbreviations: IHC, inner hair cell; SGN, spiral ganglion neuron; ROS, reactive oxygen species

Together, these agents represent an evolving pharmacological platform for auditory protection, combining neuroprotection with the potential for synaptic repair and functional restoration (Fig. 2).

#### Otoprotective, Anti-inflammatory, and Antioxidant Strategies

Corticosteroids remain first-line treatments for sudden sensorineural hearing loss (SSNHL) and autoimmune inner ear disease, acting through the suppression of inflammatory cytokines and edema (de Cates and Winters, 2025; Kim et al. 2022). However, their inability to regenerate synapses underscores the need for combined strategies (Xu et al. 2023).

Emerging anti-inflammatory agents and cytokine modulators aim to selectively inhibit NF-κB–dependent pathways and pro-inflammatory mediators (Paciello et al. 2020a, b; Manohar et al. 2022; Kang et al. 2023), while antioxidants, such as N-acetylcysteine (NAC), resveratrol, and coenzyme Q10 (CoQ10), mitigate oxidative stress and preserve cellular integrity (Fetoni et al. 2018; Muderris et al. 2022; Bai et al. 2021; Chen et al., 2001; Nunez and Guo 2025). Local delivery via intratympanic injection or drug-eluting cochlear implants offers improved bioavailability and reduced systemic toxicity compared to oral administration (Borenstein et al., 2011).

These strategies stabilize the cochlear microenvironment and limit secondary damage, thereby creating conditions that enhance neurotrophin-mediated neuroprotection and repair.

#### Gene and RNA-Based Therapies for Cochlear Synaptopathy Repair and Hearing Restoration

While neurotrophin-based therapies are particularly effective for preventing cochlear damage and protecting against early synaptic dysfunction, gene therapy offers a targeted approach for correcting genetic mutations that directly cause cochlear synaptopathy.

Mutations in OTOF, SLC17A8, and AIFM1 are prime targets for intervention, as these genes encode critical components of neurotransmitter release and synaptic vesicle cycling (Wang et al. 2023; Zhang et al., 2023; Lv et al. 2024). Delivery techniques, such as canalostomy, enable localized administration of therapeutic vectors directly into the cochlea, minimizing systemic effects and improving targeting precision (Ji et al. 2019).

Alongside gene-based strategies, cochlear implants are being enhanced with innovative technologies designed to improve their effectiveness. For example, optogenetic stimulation is being studied as a method to control neuronal activity using light, to improve cochlear implant performance by restoring neural responses. Another emerging approach is the development of drug-releasing electrodes to deliver therapeutic compounds directly to the cochlea, ensuring continuous local treatment and promoting synaptic repair.

RNA-based therapies, including microRNA and small interfering RNA (siRNA), are also being investigated for their ability to modulate gene expression in the cochlea. These therapies offer a way to regulate the expression of specific genes involved in cochlear degeneration. Studies suggest that targeting these pathways with RNA-based therapies could help prevent cochlear cell death and slow the progression of hearing loss (Hussain et al., 2024).

These novel gene-based therapies, in combination with advancements in CIs and other regenerative technologies, represent a new frontier in the treatment of CS and hearing loss. As research advances, the integration of gene-based and pharmacological therapies may pave the way for personalized and highly effective strategies for hearing restoration, offering renewed hope for individuals affected by hearing loss.

A conceptual overview of the major risk factors, pathogenic cascades, clinical manifestations, and potential therapeutic intervention points is illustrated in Fig. 2.

### Optic Neuropathy

**Fig. 3:**
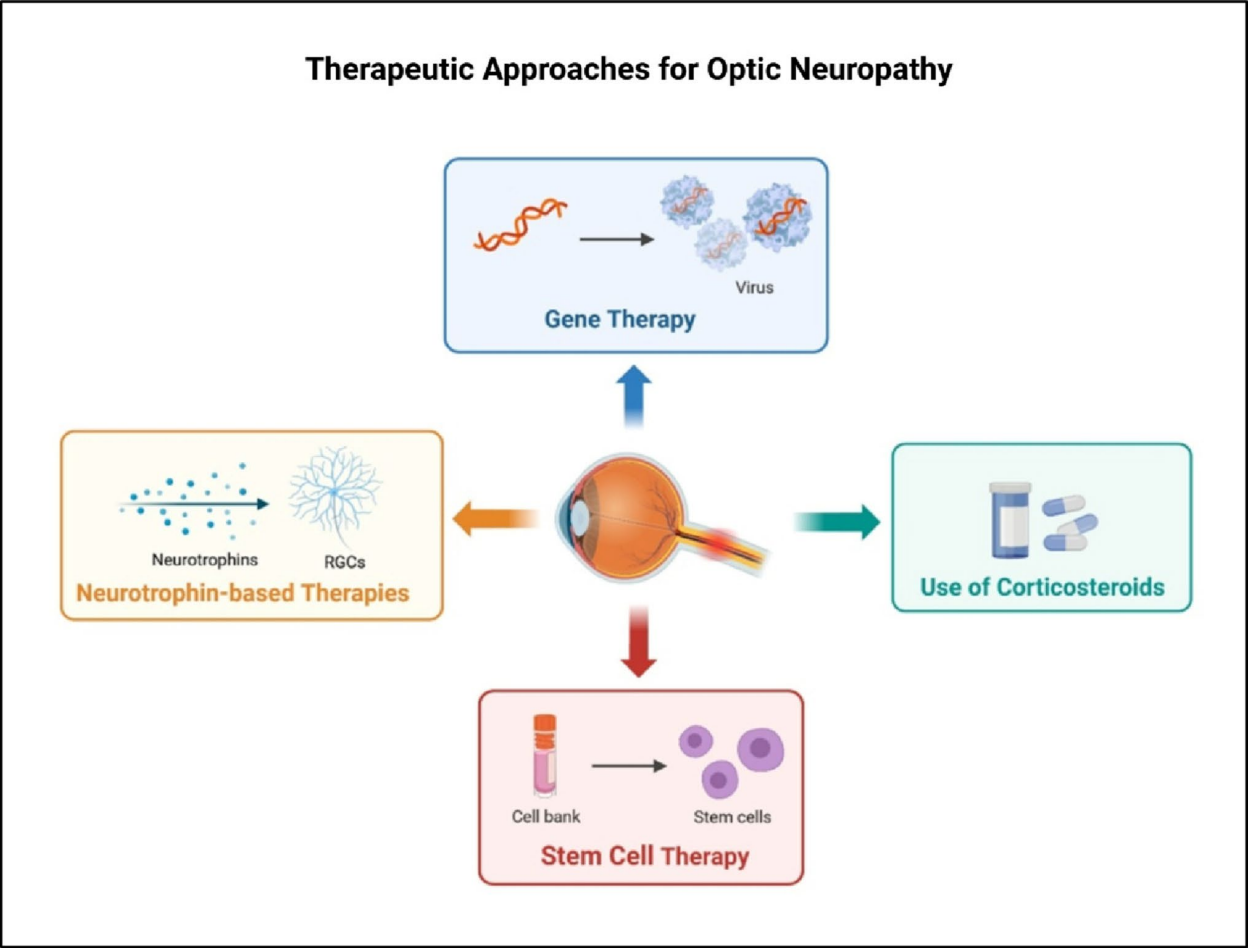
Therapeutic strategies for optic neuropathy: schematic overview of the principal therapeutic approaches currently under investigation for the treatment of optic neuropathy. Neurotrophin-based therapies (left) target retinal ganglion cells (RGCs) to promote neuroprotection, axonal regeneration, and synaptic stability. Gene therapy (top) employs viral vectors, such as adeno-associated viruses, to deliver or correct defective genes involved in mitochondrial or synaptic dysfunction. Stem cell therapy (bottom) aims to restore visual function by providing trophic support and, potentially, replacing damaged RGCs. Corticosteroid treatment (right) remains a standard option for inflammatory and demyelinating forms, primarily serving to suppress inflammation and stabilize disease progression. These complementary strategies underscore the shift toward integrative, neuroprotective, and regenerative approaches for optic neuropathy

Optic neuropathies are a heterogeneous group of disorders with limited therapeutic options, as current treatments such as corticosteroids may delay progression but rarely restore neural damage or visual function. In this setting, neurotrophins represent a biologically grounded strategy to preserve retinal ganglion cells and promote optic nerve repair, although their clinical application is still constrained by delivery, dosing, and durability issues. Emerging gene-therapy and stem-cell-based approaches may help overcome these barriers, opening new avenues for meaningful regeneration and visual recovery (Fig. 3).

Comparative evaluation of these strategies provides critical insight into their mechanisms of action, therapeutic windows, and translational potential, highlighting opportunities for combined treatments that may enhance neuroprotection and functional recovery in optic neuropathy.

#### Intraocular Pressure-Lowering Therapies

Intraocular pressure (IOP)–lowering remains the primary therapeutic strategy of managing glaucoma and related optic neuropathies, and reduction of IOP has been consistently associated with a slower rate of disease progression and reduced risk of visual field loss (Peeters et al., 2010). Pharmacological therapies employing topical ocular hypotensive agents, including prostaglandin analogues, beta-blockers, alpha-2 agonists, carbonic anhydrase inhibitors, and newer classes such as Rho-kinase inhibitors, work by either enhancing aqueous humor outflow through trabecular and uveoscleral pathways or suppressing aqueous production; randomized trials and meta-analyses demonstrate that Rho-kinase inhibitors produce IOP reductions comparable to timolol, although with a different side-effect profile (Wu et al., 2021). When medical therapy fails to achieve target intraocular pressure, laser interventions such as selective laser trabeculoplasty are widely employed to enhance trabecular meshwork outflow. Owing to their efficacy and favorable safety profile, these procedures can be used both as first-line and adjunctive treatments. Surgical and microinvasive approaches, including trabeculectomy, glaucoma drainage devices, and minimally invasive procedures, offer additional means to lower IOP in more advanced or refractory cases, often in combination with other modalities to address both mean IOP and diurnal fluctuation for optimal neuroprotection (Micheletti et al., 2025).

Although these strategies do not directly promote neuronal regeneration, they reduce mechanical and metabolic stress on retinal ganglion cells, thereby creating conditions that may enhance neurotrophin-mediated neuroprotection.

#### Conventional Therapies

Corticosteroids remain the first-line therapy for inflammatory optic neuritis, exerting anti-inflammatory and anti-edematous effects that accelerate visual recovery (Fig. 3). However, they do not prevent long-term retinal ganglion cell loss or promote axonal regeneration. These mechanistic limitations underscore the role of corticosteroids as acute, disease-modifying agents rather than definitive neuroprotective or restorative therapies. In acute optic neuritis, high-dose intravenous corticosteroids, such as methylprednisolone, accelerate visual recovery but do not significantly improve long-term visual outcomes; on this basis, they remain the standard acute treatment (Mackay et al., 2015). The clinical benefits of corticosteroids are offset by their frequent adverse effects, including immunosuppression, metabolic disturbances, and ocular complications. Importantly, glucocorticoids do not promote direct synaptic or RGC regeneration, positioning them primarily as disease-stabilizing rather than regenerative agents (Stunkel et al., 2018). In cases refractory to conventional corticosteroid therapy, monoclonal antibodies targeting the interleukin-6 receptor or other immunomodulatory agents such as methotrexate and cyclophosphamide may be employed as adjunctive treatments (Bennett et al., 2023).

Infectious optic neuritis is typically managed with antimicrobial agents directed at the specific pathogen. Nutritional optic neuritis, caused by a deficiency in essential vitamins, most commonly vitamin B12, is treated through vitamin B12 supplementation and dietary modifications to increase intake of vitamin B-rich foods. The therapeutic options for toxic optic neuropathies vary depending on the underlying causative factor, but the primary intervention is prompt discontinuation of exposure to the harmful substance.

These approaches primarily target upstream disease mechanisms but do not directly address neuronal survival and regeneration, underscoring the need for strategies that enhance intrinsic repair processes, such as neurotrophin-based interventions.

#### Neuroprotective Agents

Neuroprotective agents, including calcium channel blockers, antioxidants, and bioactive compounds, have the ability to reduce excitotoxic stress, curb inflammatory injury, and enhance the survival of RGCs. Increasing attention has been directed toward erythropoietin (EPO), whose neuroprotective and neurotrophic properties have been demonstrated in various ocular diseases (Lai et al., 2022). EPO appears to prevent neuronal apoptosis by activating key intracellular pathways, including STAT5, MAPK, PI3K/Akt, and NF-κB, while also upregulating anti-apoptotic proteins such as Bcl-xL and Bcl-2 (Chen et al., 2025). Concurrently, it can suppress pro-apoptotic mediators like µ-calpain, Bax, and caspases 8 and 9, thereby limiting RGC degeneration, enhancing neuronal viability, and shielding neural tissue from mechanical injury (Si et al., 2019). Additional compounds with neuroprotective potential include clemastine, an antihistamine with remyelinating effects (Liu et al., 2024), and brimonidine, a selective α-adrenergic agonist known for its neuroprotective and anti-inflammatory actions (Li et al., 2025). Calcium channel antagonists such as nilvadipine have also been shown to mitigate optic nerve damage in a mouse ocular hypertension model (Tsuruga et al., 2023). Idebenone, a synthetic analogue of coenzyme Q10, became the first disease-specific antioxidant approved by the European Medicines Agency in 2015 for treating the subacute or dynamic stages of Leber’s hereditary optic neuropathy (LHON). Recently, it has received FDA Priority in the US, with a decision expected by February 2026. Its suggested mechanism of action includes improving mitochondrial function and reducing retinal ganglion cell death (Ophthalmology Times 2025).

Other antioxidant bioactive compounds, including nicotinamide, pyrroloquinoline quinone, and berberine, are gaining attention as potential neuroprotective therapies for optic neuropathies, especially glaucoma. By targeting pathways implicated in glaucomatous neurodegeneration, such compounds may help preserve optic nerve integrity and slow the progression of vision loss, making them attractive candidates for adjunctive treatment in future therapeutic strategies. These agents provide indirect neuroprotection and may act synergistically with neurotrophins by improving cellular resilience and responsiveness to trophic signaling.

#### Gene and Cell-Based Therapies

Gene and stem cell therapies are being explored for their ability to modify disease progression. A notable milestone has been achieved with gene therapy for LHON (Battista et al., 2024). Because of its easy accessibility, immune-privileged environment, and relative isolation from other organs, the eye is particularly well-suited for gene-based interventions. As a result, gene therapy has emerged as a compelling avenue for precisely delivering therapeutic genetic material to specific intraocular tissues (Xu et al., 2023).

Lenadogene nolparvovec (GS010) is a recombinant adeno-associated viral vector of serotype 2 (rAAV2/2) carrying the wild-type ND4 gene (rAAV2/2-ND4), which is mutated in Leber hereditary optic neuropathy (McGrady et al., 2023) and is engineered to target the nuclei of RGCs. Multiple clinical trials (NCT02064569, NCT02652780, NCT02652767) have evaluated the safety, tolerability, and efficacy of intravitreal GS010 administration in patients with LHON. Findings indicate a favorable long-term safety profile and bilateral visual improvement following treatment with lenadogene nolparvovec (Biousse et al., 2021).

Although gene therapy has made considerable progress in ophthalmology in recent years, there are still some limitations to overcome, including defects in gene editing techniques, host immune reactions, limited efficacy in advanced disease, regulatory concerns, and elevated costs (Xu et al., 2023). Stem cell-based therapy has emerged as a compelling therapeutic alternative (Fig. 3), with the potential to prevent RGC degeneration, promote axonal regeneration, and replace lost RGCs with healthy stem-cell-derived RGCs or their precursors. Neuroprotective effects of intravitreal mesenchymal stem cell transplantation were seen in a rat model of glaucoma. This supports a neuroprotective mechanism mediated by local MSC delivery (da Silva, 2021). Mechanistically, a recent study demonstrated that a specific microRNA (microRNA-21-5p) derived from induced pluripotent stem cells (iPSCs) exerts protective effects on RGCs in a mouse model of optic nerve injury (Xia, 2025b). The proposed mechanisms include downregulation of pro-apoptotic and inflammatory genes, suppression of TNF-α-mediated inflammatory pathways, and modulation of immune responses, contributing to neuroprotection. Early clinical reports (on small cohorts) show relative safety and some functional improvements (vision, sensitivity), but no conclusive proof of halting or reversing structural degeneration (Pastor, 2023; Limoli, 2021).

The combination of neurotrophin-mediated neuroprotection and stem cell–based regeneration may offer a valuable translational strategy for RGC degeneration. Preclinical and early-stage clinical trials indicate that integrating neurotrophin delivery with stem cell transplantation (or stem cell-conditioned environments) can provide both neuroprotection and regenerative support. Recent mechanistic evidence suggests that stem cells respond to signals from injured RGCs by secreting factors that enhance survival, creating a self-reinforcing neuroprotective microenvironment (Xia, 2025a). A clinical trial (NCT01920867) evaluating autologous bone marrow-derived stem cells (BMSCs) demonstrated safety and potential visual improvement (Weiss et al., 2015). Despite these advances, key challenges remain, including optimizing stem cell sourcing and differentiation, improving delivery and integration, and defining the optimal timing and dosage to achieve both neuroprotection and functional recovery. As illustrated in the Fig. 3, gene- and cell-based approaches may serve as powerful platforms to deliver or amplify neurotrophin signaling, representing a key step toward effective regenerative therapies.

### Olfactory Neuropathy

While neurotrophins form the biological cornerstone of olfactory regeneration, complementary strategies target inflammation, oxidative stress, cellular repair, and sensory reactivation. These approaches rarely achieve complete neural restoration on their own but play a pivotal role in stabilizing the neuroimmune environment, reducing secondary damage, and enhancing the efficacy of neurotrophin-based therapies.

#### Anti-Inflammatory and Immunomodulatory Approaches

**Fig. 4:**
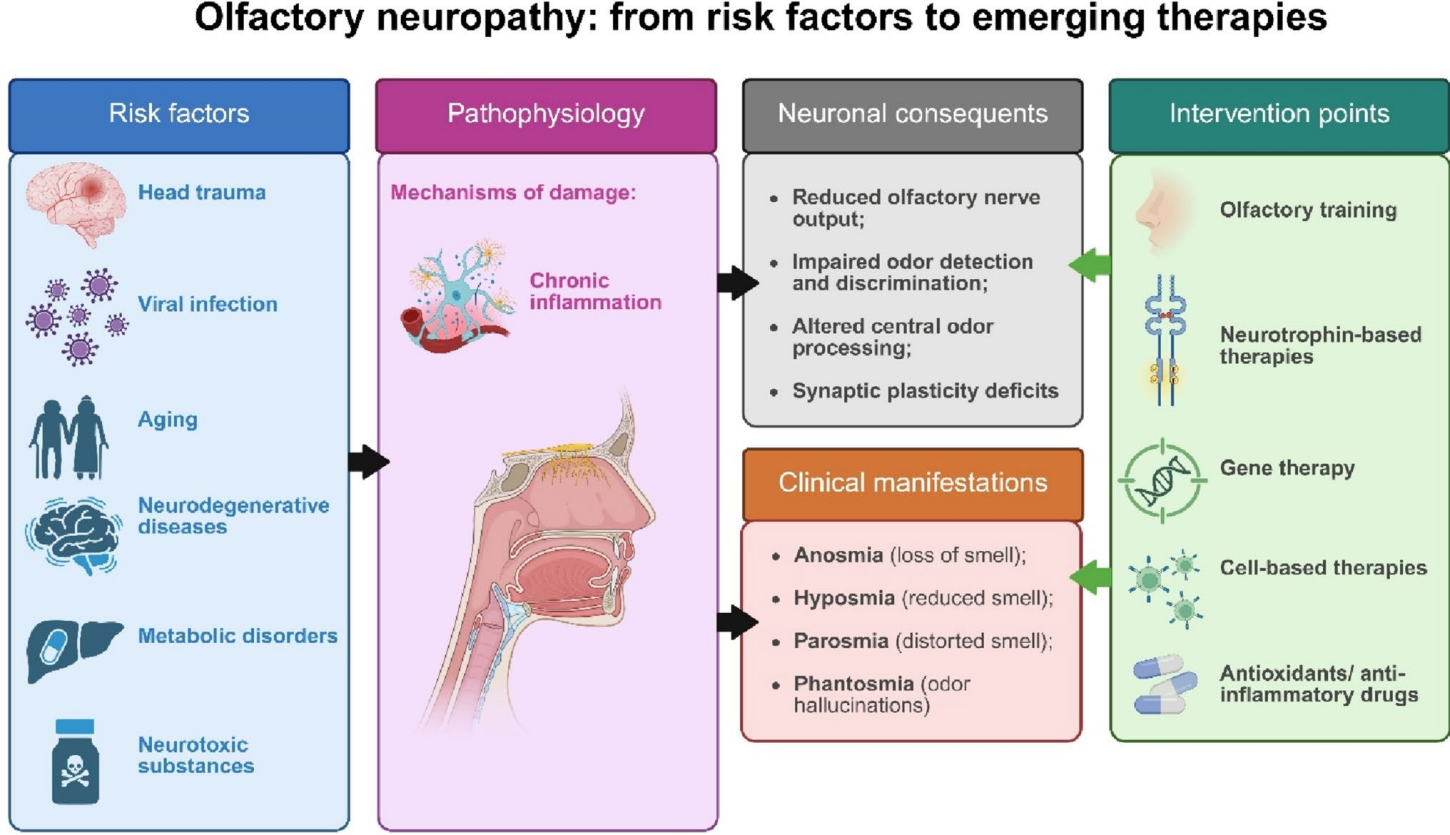
Olfactory neuropathy: risk factors, pathophysiology, and therapeutic intervention points. This diagram summarizes the primary risk factors, mechanisms, and therapeutic strategies for olfactory neuropathy. Key risk factors, including aging, head trauma, viral infections, neurodegenerative diseases, and exposure to neurotoxic agents, lead to chronic inflammation and metabolic stress in the olfactory epithelium and bulb. These processes impair olfactory function, causing clinical symptoms such as anosmia, hyposmia, parosmia, and phantosmia. Therapeutic approaches, such as neurotrophin-based therapies, olfactory training, and cell- and gene-based treatments, aim to restore olfactory neuron survival, synaptic connectivity, and functional recovery

Inflammation is a key driver of persistent olfactory dysfunction (Fig. 4), implicated in more than 130 associated disorders (Leon et al., 2024). Chronic rhinosinusitis (CRS), allergic rhinitis, and post-viral inflammation activate NF-κB- and TNF-α–mediated pathways that damage basal progenitor cells and suppress neurogenesis (Chen et al., 2019; Sultan et al., 2011; Pozharskaya et al., 2013).

Corticosteroids remain the first-line treatment but provide only partial neuroprotection and are limited by systemic side effects (Chen et al., 2017). Biologic therapies such as dupilumab, mepolizumab, and omalizumab, which target type 2 inflammatory cascades, have shown superior efficacy, particularly in CRS with nasal polyps (Han et al., 2021; De Santis et al., 2025; Otten et al., 2024). In selected refractory cases, pulsed ultrasound therapy and functional endoscopic sinus surgery (FESS) can further reduce inflammation and restore olfactory airflow (Nakhostin-Ansari et al., 2021).

By relieving inflammatory constraints on neural progenitors, these strategies create a permissive microenvironment that enables neurotrophin-driven regeneration.

#### Antioxidant and Metabolic Therapies

Compounds, sush as N-acetylcysteine (NAC), resveratrol, and coenzyme Q10 (CoQ10), mitigate ROS/RNS and enhance enzymatic antioxidant defenses (e.g., SOD, CAT). Preclinical data are encouraging, though clinical evidence remains heterogeneous. Optimized intranasal formulations and controlled-release systems improve local bioavailability and reduce systemic exposure.

These metabolic interventions appear most effective in combination with neurotrophins or anti-inflammatory treatments, serving as stabilizing co-therapies that enhance neuronal resilience and promote recovery.

#### Cell-Based Strategies

OECs provide trophic support, facilitate axonal growth and remyelination, and can be genetically modified to overexpress BDNF or NGF, amplifying local neurotrophic signaling (Feron et al., 2008). Autologous transplantation of olfactory tissue or OECs has demonstrated regenerative potential not only within the olfactory system but also in other neural circuits, such as the optic nerve (Shkarubo et al., 2020).

Key challenges include standardization of cell sources, long-range integration, and scalability under GMP conditions. Nevertheless, cell-based therapies, especially when combined with neurotrophins or biomaterial scaffolds, represent a promising avenue for restoring connectivity in severe or chronic olfactory neuropathy.

#### Olfactory Training

Olfactory training (OT) is a low-risk, activity-dependent intervention that exploits neural plasticity to improve odor threshold, discrimination, and identification. OT upregulates plasticity-related genes (BDNF, NGF, NGFR, GFAP) and, in both preclinical and clinical models, has shown superior recovery compared to corticosteroids (Kim et al., 2019; Feron et al., 2008).

Its non-invasive nature, broad applicability, and ability to synergize with neurotrophin delivery (e.g., intranasal or biomaterial-assisted administration) make OT an ideal complementary approach to consolidate synaptic remodeling and bulb connectivity.

In olfactory neuropathy, anti-inflammatory and metabolic interventions stabilize the neural niche, cell-based strategies restore structural integrity, and olfactory training enhances functional plasticity. When combined with neurotrophin-centered approaches, these modalities provide a biologically coherent framework for durable olfactory repair and sensory recovery.

In conclusion, these findings show that effective olfactory repair requires an integrated strategy in which inflammation control, metabolic support, structural regeneration, and activity-dependent plasticity converge to enhance neurotrophin signaling. In this sense, the olfactory system provides a clear example of how a permissive microenvironment can unlock the regenerative potential of neurotrophins. Accordingly, these approaches should be viewed not as alternatives, but as complementary interventions that strengthen neurotrophin-based repair by optimizing the conditions for neuronal recovery.

A conceptual overview of the principal risk factors, pathogenic cascades, clinical consequences, and therapeutic intervention points in olfactory neuropathy is illustrated in Fig. 4.

## Comparative Perspective Across Sensory Systems

**Fig. 5:**
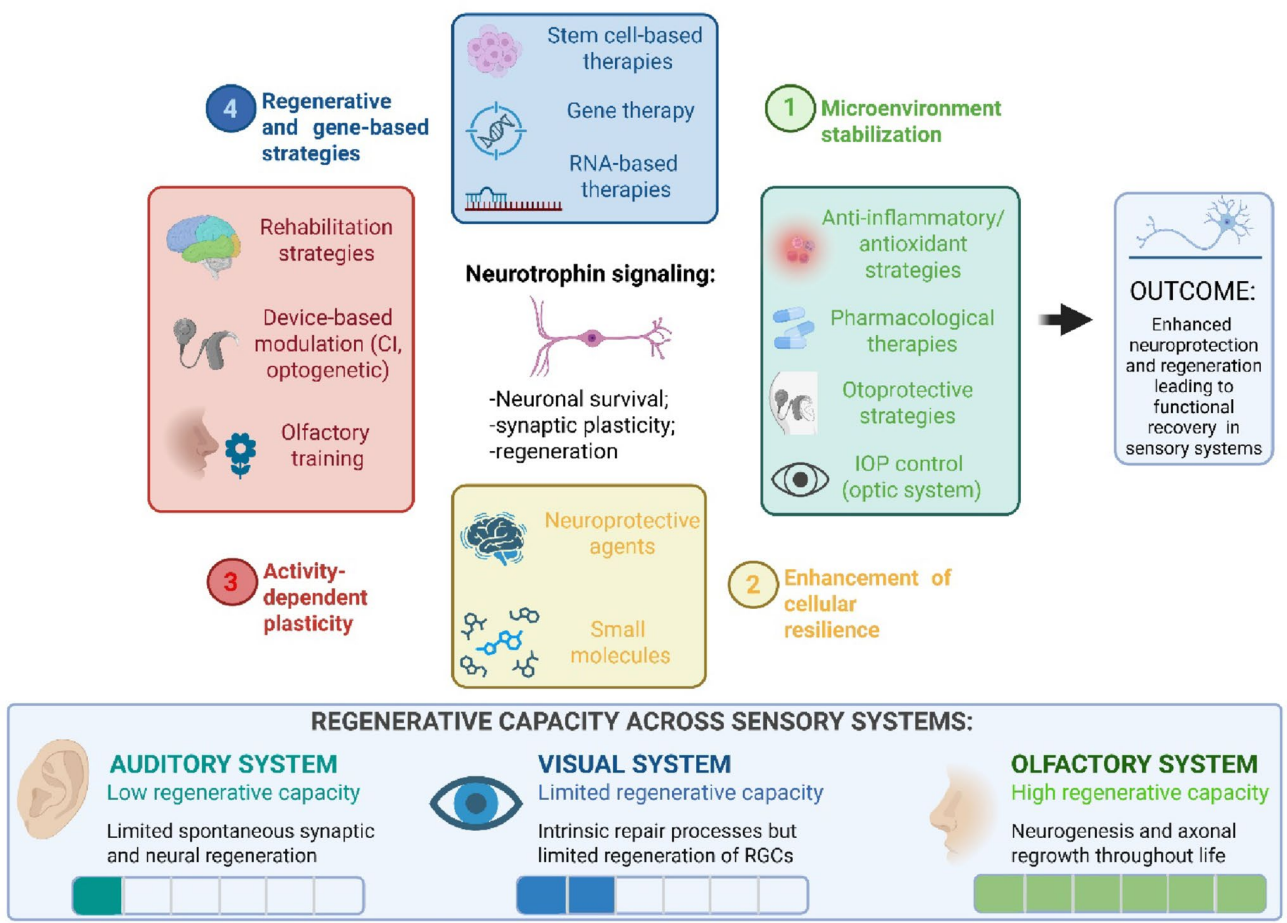
Comparative regenerative capacity and integrated therapeutic framework across sensory systems. The figure illustrates differences in intrinsic regenerative capacity and highlights a multimodal therapeutic framework centered on neurotrophin signaling, integrating microenvironment stabilization, cellular resilience, activity-dependent plasticity, and regenerative/gene-based strategies to promote neuroprotection, regeneration, and functional recovery

Although the auditory, visual, and olfactory systems share common neurotrophin-dependent mechanisms, they exhibit significant differences in terms of regenerative capacity and therapeutic response. These differences underscore the need to consider neurotrophin signaling within a cellular and microenvironmental context specific to each system (Fig. 5).

A key distinguishing feature is the intrinsic regenerative potential of each system. The olfactory epithelium maintains continuous neurogenesis supported by resident progenitor cells, a permissive extracellular matrix, and continuous neurotrophic support. In contrast, the auditory and visual systems are characterized by limited cell turnover, the absence of active progenitor populations, and a more restrictive microenvironment, which together limit regenerative processes.

Although the main neurotrophin signaling pathways, such as Trk receptor activation and the downstream PI3K/Akt and MAPK/ERK cascades, are largely conserved across all sensory systems, their functional outcomes appear to depend largely on the context. In the cochlea and retina, neurotrophin signaling primarily promotes neuronal survival and slows degeneration, whereas in the olfactory system, it actively supports neuronal renewal and circuit reorganization.

Differences in cellular architecture and tissue organization further influence the therapeutic response. The highly specialized and compartmentalized structure of the cochlea and retina, combined with limited accessibility and barriers to molecular diffusion, limits the efficacy of exogenous neurotrophin administration. In contrast, the olfactory system benefits from direct environmental exposure, greater accessibility, and dynamic interactions between neurons and glia, thereby facilitating both endogenous and exogenous regenerative signaling.

These observations support a unifying model in which neurotrophin signaling is common to all sensory systems, but its regenerative outcome is determined by the interaction between intrinsic cellular competence and the surrounding microenvironment. Understanding these system-specific constraints will be essential for developing effective therapeutic strategies aimed at restoring sensory function.

## Conclusions

Neurotrophins, including NGF, BDNF, and NT-3, play a central role in neuronal survival, synaptic plasticity, and regenerative processes within sensory systems, supporting their potential as therapeutic agents in auditory, visual, and olfactory neuropathies.

Beyond their individual roles, accumulating evidence supports a unifying framework in which neurotrophin signaling represents a conserved biological axis linking neuronal vulnerability and repair across sensory pathways. Within this perspective, auditory and visual systems exemplify conditions in which neurotrophic signaling is preserved, but regenerative capacity remains limited, whereas the olfactory system demonstrates how a permissive microenvironment can sustain effective neurotrophin-driven regeneration. This cross-system comparison highlights that the outcome of neurotrophin signaling depends not only on its presence but also on tissue-specific responsiveness and regenerative competence.

Although robust preclinical evidence supports the neuroprotective and regenerative potential of neurotrophins, clinical translation remains incomplete. Future efforts should focus on improving delivery strategies, enhancing target engagement, and integrating neurotrophin-based therapies with complementary approaches, including gene therapy, cell-based interventions, and activity-dependent rehabilitation.

Ultimately, neurotrophin-centered strategies, particularly when combined with approaches that modulate the tissue microenvironment and intrinsic regenerative capacity, may offer a path toward true disease modification and durable restoration of sensory function.

Future research should aim to optimize delivery methods and to incorporate neurotrophin therapies with complementary treatments. By addressing the underlying causes of sensory neuropathies while promoting regenerative processes, neurotrophin-based therapies have the potential to achieve sustained sensory recovery and provide disease-modifying effects.

## Limitations

This review has some limitations. Much of the available evidence is derived from preclinical studies, and therefore, the clinical applicability of neurotrophin-based strategies has yet to be fully established.

Although early translational and clinical studies suggest potential benefit in selected settings, efficacy has often been partial or variable, particularly in advanced disease stages, where structural degeneration may limit the restorative effects of trophic support alone (Lambiase et al., 2005; Beykin et al., 2022).

In addition, the translation of neurotrophins into clinical practice is constrained by pharmacokinetic and delivery-related challenges, including short half-life, rapid degradation, and limited tissue penetration across biological barriers such as the blood–retina and blood–labyrinth barriers (Fu et al., 2019; Plontke et al., 2017). While recent advances in localized and sustained delivery systems, such as nanoparticle-based platforms, gene therapy approaches, and controlled-release technologies, offer potential solutions, their long-term safety and efficacy still require further validation.

Neurotrophin-based approaches may also be limited by adverse effects and context-dependent signaling. For example, NGF administration has been associated with pain and hyperalgesia in clinical studies, restricting its systemic use (Petty et al., 1994). In addition, activation of receptors such as p75^NTR^ may produce divergent or even maladaptive effects depending on the cellular environment, underscoring the need for precise spatial and temporal control of neurotrophin signaling.

Furthermore, advanced therapeutic platforms designed to sustain neurotrophin delivery, including gene- and cell-based strategies, raise additional challenges related to manufacturing cost, regulatory complexity, scalability, and broad clinical accessibility.

Finally, given the rapid evolution of the field, emerging molecular targets and therapeutic strategies not covered in this review may further refine and expand current perspectives.

## Data Availability

No datasets were generated or analysed during the current study.
